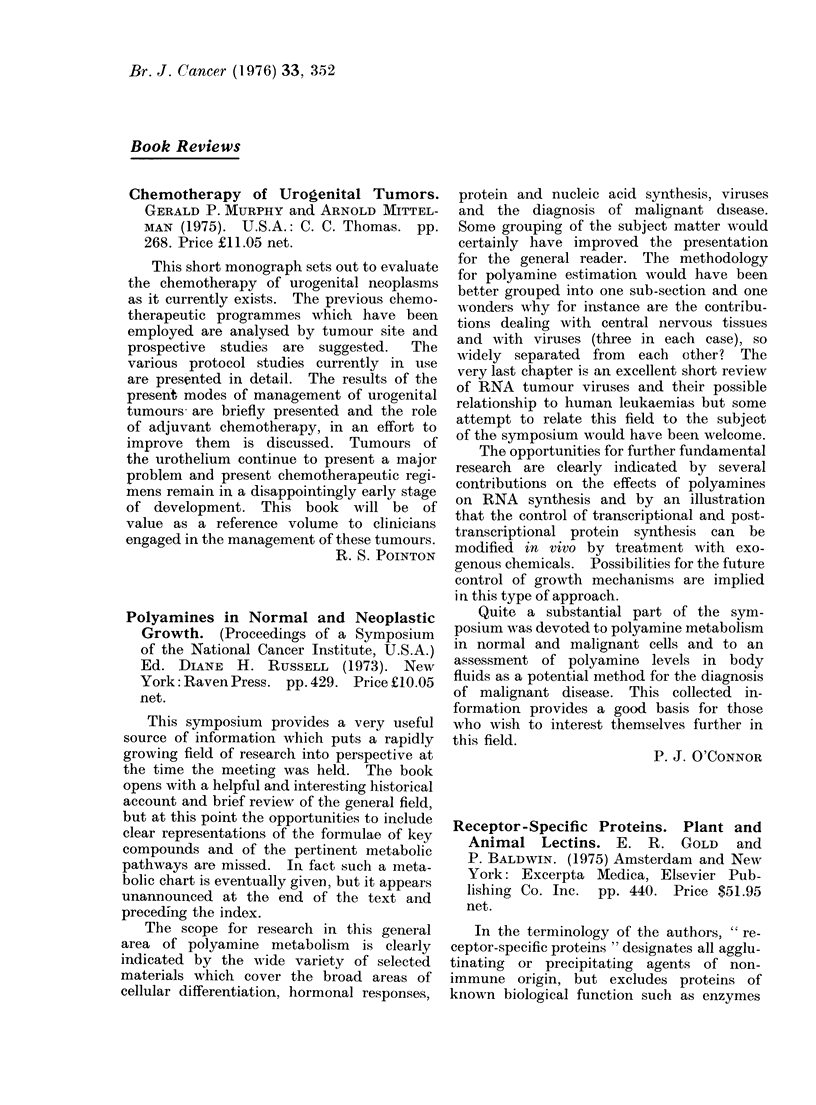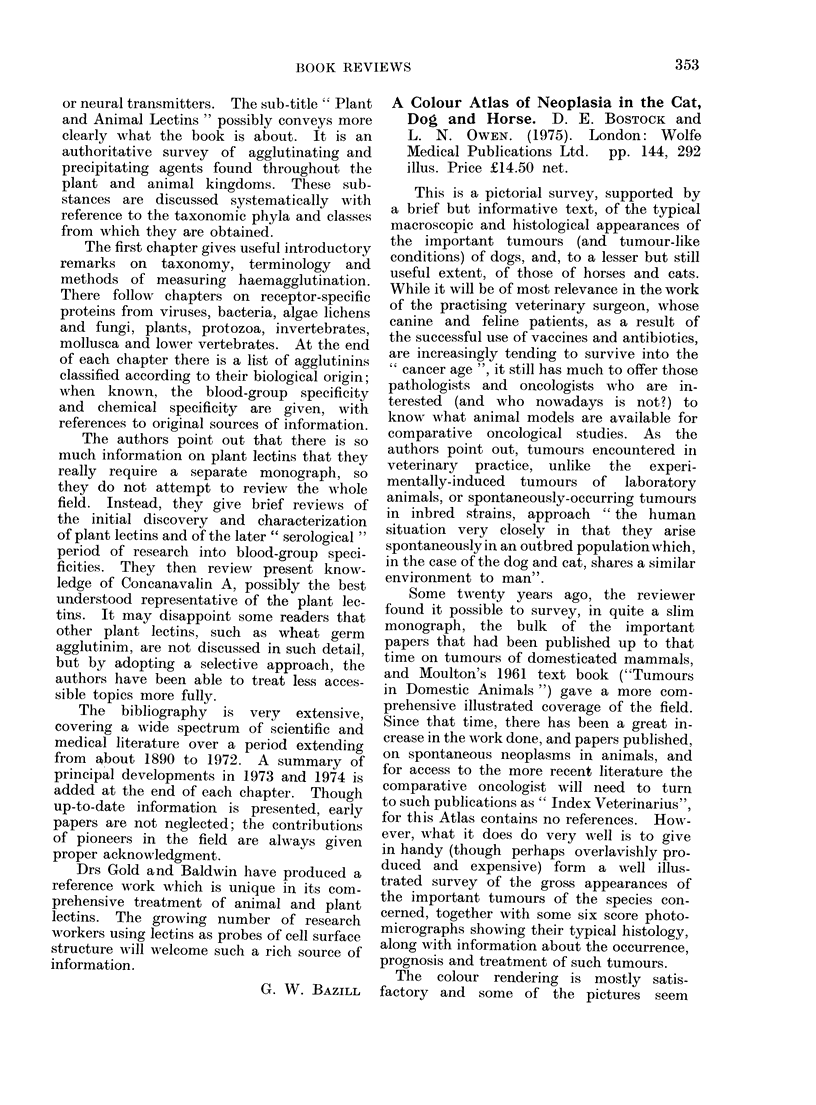# Receptor-Specific Proteins. Plant and Animal Lectins

**Published:** 1976-03

**Authors:** G. W. Bazill


The scope for research in this general area of polyamine metabolism is clearly indicated by the wide variety of selected materials which cover the broad areas of cellular differentiation, hormonal responses, protein and nucleic acid synthesis, viruses and the diagnosis of malignant disease. Some grouping of the subject matter would certainly have improved the presentation for the general reader. The methodology for polyamine estimation would have been better grouped into one sub-section and one wonders why for instance are the contributions dealing with central nervous tissues and with viruses (three in each case), so widely separated from each other? The very last chapter is an excellent short review of RNA tumour viruses and their possible relationship to human leukaemias but some attempt to relate this field to the subject of the symposium would have been welcome.

The opportunities for further fundamental research are clearly indicated by several contributions on the effects of polyamines on RNA synthesis and by an illustration that the control of transcriptional and post-transcriptional protein synthesis can be modified *in vivo* by treatment with exogenous chemicals. Possibilities for the future control of growth mechanisms are implied in this type of approach.

Quite a substantial part of the symposium was devoted to polyamine metabolism in normal and malignant cells and to an assessment of polyamine levels in body fluids as a potential method for the diagnosis of malignant disease. This collected information provides a good basis for those who wish to interest themselves further in this field.

P. J. O'CONNOR

**Receptor-Specific Proteins. Plant and Animal Lectins.** E. R. GOLD and P. BALDWIN. (1975) Amsterdam and New York: Excerpta Medica, Elsevier Publishing Co. Inc. pp. 440. Price $51.95 net.

In the terminology of the authors, " receptor-specific proteins " designates all agglutinating or precipitating agents of non-immune origin, but excludes proteins of known biological function such as enzymes or neural transmitters. The sub-title " Plant and Animal Lectins " possibly conveys more clearly what the book is about. It is an authoritative survey of agglutinating and precipitating agents found throughout the plant and animal kingdoms. These substances are discussed systematically with reference to the taxonomic phyla and classes from which they are obtained.

The first chapter gives useful introductory remarks on taxonomy, terminology and methods of measuring haemagglutination. There follow chapters on receptor-specific proteins from viruses, bacteria, algae lichens and fungi, plants, protozoa, invertebrates, mollusca and lower vertebrates. At the end of each chapter there is a list of agglutinins classified according to their biological origin; when known, the blood-group specificity and chemical specificity are given, with references to original sources of information.

The authors point out that there is so much information on plant lectins that they really require a separate monograph, so they do not attempt to review the whole field. Instead, they give brief reviews of the initial discovery and characterization of plant lectins and of the later " serological " period of research into blood-group specificities. They then review present knowledge of Concanavalin A, possibly the best understood representative of the plant lectins. It may disappoint some readers that other plant lectins, such as wheat germ agglutinim, are not discussed in such detail, but by adopting a selective approach, the authors have been able to treat less accessible topics more fully.

The bibliography is very extensive, covering a wide spectrum of scientific and medical literature over a period extending from about 1890 to 1972. A summary of principal developments in 1973 and 1974 is added at the end of each chapter. Though up-to-date information is presented, early papers are not neglected; the contributions of pioneers in the field are always given proper acknowledgment.

Drs Gold and Baldwin have produced a reference work which is unique in its comprehensive treatment of animal and plant lectins. The growing number of research workers using lectins as probes of cell surface structure will welcome such a rich source of information.

G. W. Bazill